# The ABCDE approach in critically ill patients: A scoping review of assessment tools, adherence and reported outcomes

**DOI:** 10.1016/j.resplu.2024.100763

**Published:** 2024-09-19

**Authors:** Laura J. Bruinink, Marjolein Linders, Willem P. de Boode, Cornelia R.M.G. Fluit, Marije Hogeveen

**Affiliations:** a Radboud University Medical Center, Amalia Children’s Hospital, Department of Paediatrics, Nijmegen, The Netherlands; b Radboud University Medical Center, Amalia Children’s Hospital, Department of Paediatrics, Division of Neonatology, Nijmegen, The Netherlands; c Radboud University Medical Center, Radboudumc Health Academy, Nijmegen, The Netherlands

**Keywords:** ABCDE, Airway Breathing Circulation Disability Exposure, Primary survey, Assessment, Adherence

## Abstract

**Aim:**

The systematic Airway, Breathing, Circulation, Disability, and Exposure (ABCDE) approach is a priority-based consensus approach for the primary assessment of all categories of critically ill or injured patients. The aims of this review are to provide a wide overview of all relevant literature about existing ABCDE assessment tools, adherence to the ABCDE approach and related outcomes of teaching or application of the ABCDE approach by healthcare professionals.

**Methods:**

A comprehensive scoping review was conducted following the Joanna Briggs Institute guidelines and reported according to the PRISMA-ScR Checklist. An a priori protocol was developed. In March 2024, MEDLINE, EMBASE, CINAHL and Cochrane library were searched to identify studies describing healthcare professionals applying the ABCDE approach in either simulation settings or clinical practice. Two reviewers independently screened records for inclusion and performed data extraction.

**Results:**

From n = 8165 results, fifty-seven studies met the inclusion criteria and reported data from clinical care (n = 27) or simulation settings (n = 30). Forty-two studies reported 39 different assessment tools, containing 5 to 36 items. Adherence to the approach was reported in 43 studies and varied from 18–84% in clinical practice and from 29–35% pre-intervention to 65–97% post-intervention in simulation settings. Team leader presence and attending simulation training improved adherence. Data on patient outcomes were remarkably scarce.

**Conclusion:**

Many different tools with variable content were identified to assess the ABCDE approach. Adherence was the most frequently reported outcome and varied widely among included studies. However, association between the ABCDE approach and patient outcomes is yet to be investigated.

## Introduction

The Airway, Breathing, Circulation, Disability, Exposure (ABCDE) approach is a systematic approach for the primary survey of all categories of critically ill or injured patients.^1^ Initially, the ABCDE approach was developed to improve trauma care, but nowadays it is used in all potential medical emergencies and applicable to patients of all ages.^2^, ^3^ The ABCDE approach is advocated to be a universal tool with the aim to assess and treat patients conform the ‘treat first what kills first’ principle. However, ABCDE algorithms and assessment tools differ amongst studies and life support courses.^4^, ^5^, ^6^, ^7^, ^8^, ^9^ Several studies and personal observations suggested that adherence to the ABCDE approach varies between healthcare professionals.^4^, ^5^ Variations in algorithms and suboptimal adherence to this approach might hypothetically affect patient outcomes. The ABCDE approach is based on expert consensus and reviews on adherence to the ABCDE approach specifically or related outcomes could not be identified. As the ABCDE approach is recommended by (inter)national life support courses and guidelines and a majority of healthcare professionals is ABCDE trained,^6^, ^7^, ^8^, ^9^ insight into adherence and the impact on outcomes is of importance for every healthcare professional possibly encountering critically ill or injured patients. With the purpose to identify research into the ABCDE approach and its outcomes, a scoping review was considered the most suitable approach.^10^, ^11^ The objectives of this study are to provide an overview of 1) all relevant literature about existing ABCDE assessment tools, 2) reported adherence to the ABCDE approach (completeness and/or correct order) and influencing factors, and 3) other professional, team or patient related outcomes of teaching and application of the ABCDE approach by healthcare professionals in a hospital setting.

## Methods

This scoping review follows the guidelines of the Joanna Briggs Institute (JBI) manual.^12^ The PRISMA-ScR Checklist was used to document the selection process and is attached in Appendix A.^13^ As recommended by Peters et al.,^14^ an a priori protocol was developed and published in the Open Science Framework.^15^

### Eligibility criteria

The following eligibility criteria were applied:

*Participants:* healthcare professionals (nurses, nurse practitioners, physician assistants, residents, medical specialists) or healthcare students.

*Concept:* the ABCDE approach and its application in clinical practice or in a simulation setting. Since the ABCDE approach is part of the primary survey, studies reporting specifically on the primary survey (without distinction of the different ABCDE domains) were considered eligible as well.

*Context:* any acute care situation in a hospital where the ABCDE approach was taught or applied in clinical practice or simulation settings (including courses).

*Study selection*: All type of studies (quantitative, qualitative, mixed-method), except reviews, were considered eligible if assessment of or adherence to the ABCDE approach, or any other outcome related to application or teaching of the ABCDE approach was described.

The following exclusion criteria were applied: conference abstracts, languages other than English, Dutch, German, French and Spanish and studies performed in a pre-hospital setting. Although the application of the ABCDE approach itself should not differ from a hospital setting, the diagnostical, therapeutical and team resources differ significantly. The literature search was not limited by year of publication.

### Information sources and search strategy

A three-step search strategy was performed as recommended by Briggs.^10^ First, an initial limited search was conducted, followed by an analysis of relevant keywords used in titles and abstracts and of index terms (MeSH terms) used to label the identified articles. Second, a complete and thorough search strategy was constructed with the assistance of an experienced literature specialist. The final search strategy is available in Appendix B. This search was executed in MEDLINE, EMBASE, CINAHL and the Cochrane library from inception until March 3, 2024. Thirdly, backward citation searching was performed on all included full text articles.

### Study selection

The search results were collected and deduplicated in EndNote X9, and subsequently imported into Rayyan (https://rayyan.qcri.org). One reviewer (LB) screened all titles and abstracts for relevance and classified the articles into two categories: ‘clearly not eligible’ (unquestionably wrong participants, wrong context and wrong concept stated in title or abstract) and ‘potentially eligible 1'. The articles in the category ‘potentially eligible 1' were independently screened on title and abstract by two reviewers (LB, ML) and classified into ‘not eligible’ and ‘potentially eligible 2'. Full-text screening was performed on all ‘potentially eligible 2' articles by LB and ML independently. Eventually, backward citation searching was conducted on all included full text articles by LB and ML. In every stage of the selection, discrepancies were solved through discussion with a third reviewer (MH).

### Data items and data charting process

Two reviewers (LB and ML) individually extracted and assessed the data of the selected full-text articles using a specifically designed pre-piloted spreadsheet, adapted from the JBI scoping review methodological guidance (Appendix D).^10^ Abstracted data included article characteristics, study aims, methods, participants, concept, context, discipline, described outcomes and the ABCDE algorithm used*.* No authors were contacted for obtaining additional data. No formal methodological quality assessment was performed given the broad scope of this review and the expected heterogeneity of the included studies.

### Synthesis of results

After data extraction, both quantitative and qualitative content analyses were conducted. A frequency analysis was performed to map the distribution of studies by year of publication, country of origin, study design, concept (ABCDE or primary survey), context (clinical practice or simulation) and discipline. Content analysis was performed by one reviewer (LB) and checked by a second reviewer (ML). Reported outcomes were categorized into three main outcome groups: 1) assessment tools, 2) adherence to the ABCDE approach and 3) other outcomes. The outcome group was divided into the following subgroups: a. professional outcomes (e.g. confidence and knowledge), b. team outcomes (such as communication and teamwork), c. patient outcomes (such as mortality and length of hospital stay), d. other outcomes.

## Results

### Selection of sources of evidence

**Fig. 1:**
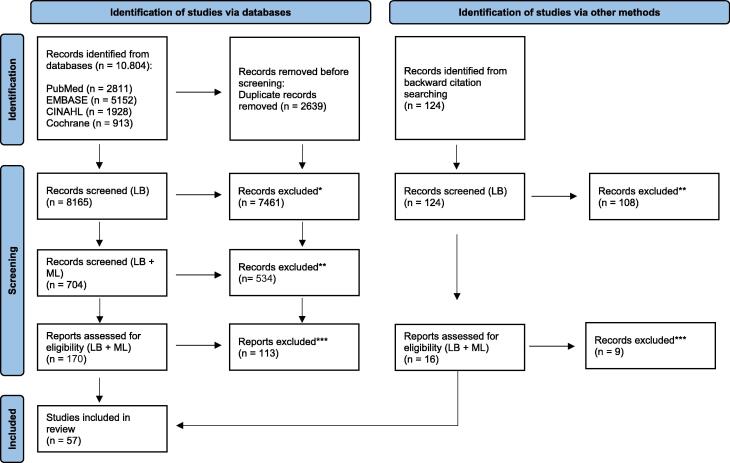
**Flow chart study selection.** * Neither participants, concept nor context stated in title or abstract. ** Wrong participants, concept or participants stated in title or abstract. *** Reasons for exclusion (wrong participants, wrong concept, wrong context) can be found in Appendix C.

The search identified 10,416 citations. After removing duplicates, screening on title and abstract, followed by full text screening and discussion, 50 studies were included (Fig. 1).^4^, ^5^, ^16^, ^17^, ^18^, ^19^, ^20^, ^21^, ^22^, ^23^, ^24^, ^25^, ^26^, ^27^, ^28^, ^29^, ^30^, ^31^, ^32^, ^33^, ^34^, ^35^, ^36^, ^37^, ^38^, ^39^, ^40^, ^41^, ^42^, ^43^, ^44^, ^45^, ^46^, ^47^, ^48^, ^49^, ^50^, ^51^, ^52^, ^53^, ^54^, ^55^, ^56^, ^57^, ^58^, ^59^, ^60^, ^61^, ^62^, ^63^ Backward citation searching identified another 7 articles eligible for inclusion,^64^, ^65^, ^66^, ^67^, ^68^, ^69^, ^70^ resulting in a total of 57 studies.

### Characteristics of sources of evidence

The majority of the included studies (n = 38, 66%) were published in the last ten years (Table 1). Most studies were conducted in Europe (n = 23, 40%) and North America (n = 23, 40%). The design varied from observational studies (n = 37, 64%); intervention studies (n = 19, 33%) of which 8 were randomized,^4^, ^21^, ^22^, ^38^, ^41^, ^67^, ^69^, ^70^ to one mixed-method study.^50^ Table 1 shows study characteristics sorted by medical discipline. Half of the studies reported data from clinical practice and half from a simulation setting. The major discipline was traumatology (n = 36, 62%), of which 21 concerned paediatric trauma. Two studies investigated the assessment of the ABCDE approach in healthy individuals.

**Table 1:** Study characteristics.

First author	Year, Country	Study design	Context	Concept	Participants/Assessments	Reported outcomes
**CLINICAL PRACTICE**						
TRAUMA						
Aukstakalnis^17^	2020, Lithuania	Observational	ED*	Primary survey	143 team assessments	Adherence: Adherence, time to completion Team: Non-technical skills
Bergs^23^	2005, The Netherlands	Observational	ED*	ABCDE†	193 team assessments	Team: Communication
Gyedu^34^	2022, Ghana	Observational	ED*	Primary survey	1006 assessments by ED health care providers (doctors, physician assistants, nurses)	Adherence: Primary assessment and actions, Reassessment
Hoff^35^	1997, USA	Observational	Trauma area*	Primary survey†	425 team assessments	Adherence: Adherence, time to completion
Koko^48^	2023, Sudan	Observational	Trauma room	ABCDE	50 team assessments	Adherence: Adherence, facilitators and barriers
Lubbert^66^	2009, The Netherlands	Observational	ED*	Primary survey	387 team assessments	Adherence: Quality appraisal for ATLS items, timing of ATLS items Team: Errors in team organization
Maluso^51^	2016, USA	Observational	ED*	ABCDE	170 team assessments	Adherence: Tasks completed at 2 min and 5 min Team: Team size, team leader performance, closed loop communication
Ritchie^57^	1999, Australia	Observational	ED	Primary survey	50 team assessments	Adherence: Time to completion, team leader performance
Spanjersberg^59^	2009, The Netherlands	Observational	ED*	ABCDE	193 team assessments	Adherence: Protocol compliance, trauma resuscitation time, timing of ATLS steps
Tsang^61^	2013, Canada	Observational	ED*	Primary survey	508 team assessments	Adherence: Compliance rate with ATLS protocols
PAEDIATRIC TRAUMA						
Botelho^24^	2020, Brazil	Observational	ED*	ABCDE	64 assessments by physicians (surgeons, surgical residents, paediatricians)	Adherence: Adherence
Botelho^25^	2021, Brazil	Interventional	ED*	ABCDE	80 assessments by physicians (surgeon, surgery resident, paediatrician)	Adherence: Adherence, time to initiate primary survey
Carter^26^	2013, USA	Observational	ED*	Primary survey	237 team assessments	Adherence: Frequency, time to completion, associated factors
Gala^31^	2016, USA	Observational	ED*	ABCD	228 assessments by paediatric or emergency medicine resident, nurse practitioner, fellow or attending	Adherence: Adherence, time to completion
Kelleher^43^	2014, USA	Interventional	ED*	ABCDE	435 team assessments	Adherence: Primary survey tasks, time to completion
Kelleher^44^	2014, USA	Interventional	ED	Primary survey	437 assessments by resident or nurse practitioner	Adherence: Model fitness, conformance, task completion sequence pattern
Kelleher^45^	2014, USA	Observational	ED	ABCDE	201 team assessments	Adherence: Task completion
O’Connell^53^	2017, USA	Observational	ED*	Primary survey	135 team assessments	Adherence: Frequency, time to completion tasks
Oakley^68^	2005, Australia	Observational	ED	ABCD	90 team assessments	Adherence: Errors
Taylor^60^	2020, USA	Interventional	ED*	Primary survey†	54 team assessments	Adherence: Time to completion
Wurster^62^	2017, USA	Observational	ED	ABCDE	142 team assessments	Adherence: Adherence, resuscitation time, timing of ATLS steps Patient: ED length of stay
Yan^63^	2020, USA	Observational	Trauma bay*	Primary survey	188 assessments by residents	Adherence: Primary survey scores
SURGERY						
Glanville^33^	2021, Australia	Interventional	Surgical department	ABCDE	74 nurses	Adherence: Adherence, time assessment
CRITICALLY ILL ADULT PATIENTS						
Olgers^5^	2017, The Netherlands	Observational	ED	ABCDE	270 assessments by attending physician, consultant, resident, medical student.	Adherence: Frequency, adherence, time to initiation, time to completion Professional: Reasons for not applying
CRITICALLY ILL PATIENTS						
Althobity^16^	2024, Saudi Arabia	Observational	University medical centre	ABCDE†	242 health care professionals (anaesthesiology, paediatrics, ED, ICU, NICU)	Professional: Knowledge
Schoeber^58^	2022, The Netherlands	Observational	University medical centre	ABCDE†	240 health care professionals (ED, anaesthesiology, paediatrics, ICU, PICU and NICU)	Professional: Knowledge ABCDE
PAEDIATRICS						
Renning^56^	2022, Malawi	Observational	Paediatric critical care units	ABCDE	153 nurses	Professional: Confidence
**SIMULATION SETTING**						
TRAUMA						
Barnes^18^	2017, Malawi	Observational	Course	ABCDE†	20 nurses	Professional: Self-reported confidence
Gillman^32^	2016, Canada	Observational	Course	Primary survey	11 teams	Adherence: Global rating score, ATLS checklist Professional: Satisfaction with course
Holcomb^36^	2002, USA	Interventional	Course	ABCD	10 assessments by 10 teams	Adherence: 5 scored tasks, 8 timed tasks, Final scores Professional: Comfortable caring for critically ill patients
Hultin^39^	2019, Sweden	Observational	Training	ABCDE	55 medical students performed 23 team assessments	Adherence: ABCDE checklist Professional: Situational Awareness Team: TEAM
Long^49^	2019, USA	Observational	Training	Primary survey†	67 assessments by multidisciplinary teams	Adherence: Time to completion primary survey
Pringle^55^	2015, Nicaragua	Interventional	Course	Primary survey	33 physicians and resident physicians	Adherence: Number of critical actions completed, time to completion Professional: Knowledge test
PAEDIATRIC TRAUMA						
Auerbach^64^	2014, USA	Interventional, Qualitative	Training	ABCD	398 health care providers performed 22 simulations	Adherence: Trauma team performance Team: Team organization
Civantos Fuentes^27^	2011, Spain	Observational	Course	Primary survey	156 paediatric primary care paediatricians	Adherence: Compliance, time
Dickerson- Young^28^	2020, USA	Observational, Qualitative	Course	ABCDE	49 participants (paediatric residents, medical students, and nurse practitioners)	Adherence: Time to completion Professional: Confidence after simulation
Falcone^30^	2008, USA	Interventional	Course	ABCD	46 scenario’s performed by 160 multidisciplinary team members	Adherence: Adherence appropriate and timely care measures, task achievement, performance in early and late simulation sessions
Holland^37^	2020, USA	Observational	Training	Primary survey†	245 paediatric residents	Professional: Confidence
Hulfish^38^	2021, USA	Interventional	Training	ABCDE	131 simulated trauma resuscitations performed by teams.	Adherence: Checklist elements completed, time to completion Professional: Mental efforts
Hunt^65^	2006, USA	Observational	Training	Primary survey	35 simulation scenarios by 35 teams	Adherence: ED tasks in need of improvement, ED tasks performed well
Parsons^69^	2014, USA	Qualitative, Interventional	Training	ABCDE	48 simulation scenarios performed by teams.	Adherence: ATLS performance score, Checklist compliance Professional: Workload during using the checklist.
CRITICALLY ILL ADULT PATIENTS						
Berg^20^	2021, Norway	Observational	Training	ABCDE†	3 scenarios by 1 emergency department team	Adherence: Overall score, clinical actions, time
Drost – de Klerck^29^	2020, The Netherlands	Observational	Course	ABCDE	30 participants: first year residents and non-residents	Adherence: Primary assessment score, skills and competences
Innocenti^40^	2022, Italy	Observational	Course	ABCDE	76 residents emergency medicine	Adherence: ABCDE assessment, ABCDE management
Jonsson^41^	2021, Sweden	Observational	Course	ABCDE†	105 participants (26 physicians, 79 nurses) in 26 teams ICU	Adherence: ABCDE Checklist Team: Leadership, teamwork, task management
Jonsson^42^	2021, Sweden	Interventional	Training	ABCDE	20 inter-professional teams, 75 nurses and physicians	Adherence: ABCDE-checklist Professional: Situation awareness Team: TEAM
Kliem^47^	2022, Switzerland	Observational	Training	ABCDE	74 residents in intensive care medicine, emergency medicine, internal medicine, and neurology	Adherence: Adherence, risk-factors non-adherence, deduced management
Macnamara^50^	2021, UK	Mixed-methods	Training	ABCDE†	20 final year medical students	Other: Simulation experience, relation to clinical practice
Merriman^67^	2014, UK	Interventional	Training	ABCDE	34 first year undergraduate nursing students	Adherence: OSCE (Objective Structured Clinical Examination) Professional: Self-efficacy and self-reported competency, Other: evaluation teaching method
Stayt^70^	2015, UK	Interventional	Training	ABCDE	98 first year nursing students	Adherence: OSCE Professional: Self-efficacy and self-reported competency, Other: Evaluation of teaching method
Peran^54^	2020, Czech Republic	Observational	Training	ABCDE	48 paramedic students	Adherence: Number performed assessment steps, order of assessment steps, time
INTERNAL MEDICINE						
Kiessling^46^	2022, Sweden	Interventional	Training	ABCDE†	123 participants: 21 physicians, 20 nurses, 14 assistant nurses, 37 medical students and 31 student nurses.	Professional: Self-efficacy
PAEDIATRICS						
Benito^19^	2018, Spain	Observational, Qualitative	Course	ABCDE†	402 paediatricians, emergency paediatricians, paediatric residents, other professionals	Professional: Application Other: Evaluation course
Nadel^52^	2000, USA	Interventional	Course	ABCD	58 paediatric residents	Adherence: Time to completion Professional: Knowledge, technical skills, experience and confidence
NEONATOLOGY						
Linders^4^	2021, The Netherlands	Interventional	Training	ABCDE	72 nurses, nurse practitioners, physician assistants, paediatric residents, neonatal fellows, neonatologists.	Adherence: Adherence
HEALTHY INDIVIDUALS						
Berg^22^	2020, Norway	Interventional	Training	ABCDE	289 medical and nursing students	Adherence: Documentation 8 ABCDE items in 5 min.
Berg^21^	2021, Norway	Interventional	Training	ABCDE	289 medical and nursing students	Adherence: Documentation 8 ABCDE items in 5 min Professional*:* Confidence
ED=Emergency Department, * Level 1 trauma center, † ABCDE or primary survey items not reported						

### Results of individual sources of evidence

1) **ABCDE assessment tools**

Of the 48 studies reporting adherence to or time to complete the ABCDE approach or primary survey, 42 studies published 39 different assessment tools. Of these 42 studies, 23 studies used the term ABCDE approach in their assessment tools, 14 studies primary survey without distinction between the different domains of the ABCDE, and five studies used an incomplete approach, i.e. ABCD (Table 1). Two studies that reported adherence,^35^, ^49^ two studies that reported time to completion,^28^, ^60^ and one study reporting both,^20^ did not elucidate which items were included in their assessment tool. The assessment tools were used during live observations or video reviews and scored team performance or individual performance (Table 2). The assessment tools were developed to 1) evaluate adherence to the ABCDE approach or primary survey, 2) investigate factors influencing adherence, 3) identify omissions during the assessment, 4) optimize team performance (leadership and optimum team size) or 5) evaluate teaching and training methods. For the 39 assessment tools used, different validation methods were identified and reported in Table 2. Sixteen tools were based on a life support course protocol (e.g. ATLS or APLS). Sixteen studies used previously published assessment tools of which two studies did not meet the inclusion criteria of this review.^71^, ^72^ Only one study reported intra-rater reliability (Intraclass Correlation Coefficient (ICC) which was 0.87 (95% Confidence Interval 0.74–0.94).^4^ Six studies assessed inter-rater reliability measured with different statistical tests, described in Table 2. Eight studies used expert consensus to compose an assessment tool. The number of items in the assessment tools ranged from 5 to 36. Nineteen assessment tools included subsequent actions such as radiology investigations, laboratory tests, and treatment such as oxygen supplementation or fluid resuscitation, which are not components of the ABCDE assessment itself. The assessment tool scores were used for improvement of a training program in two studies^17^, ^66^ and in some studies, all participants or worst performers were invited for a review of their assessment as a learning opportunity.^57^, ^60^, ^62^

2) **Adherence towards the ABCDE approach**

**Table 2:** Summary of assessment tools.

Author	Aim study	Participants		Context		Content			Observation		Validation			
		*Individual*	*Team*	*Simulation*	*Clinical care*	*ABCDE-items (N)*	*Assess-ment*	*Action*	*Video*	*Live*	*Protocol based*	*Published tools*	Reliability ((ICC/κ/r/ρ)*)	*Expert consensus*
Auerbach^64^	Measure impact of a quality improvement simulation program		✓	✓		11	✓			✓				
Aukstakalnis^17^	Performance analysis and feedback		✓		✓	11	✓		✓		✓ATLS			
Berg^22^	Non-inferiority of individual VR versus traditional equipment	✓		✓		8	✓		✓					
Berg^21^	Non-inferiority of multiplayer VR versus traditional equipment	✓		✓		8	✓		✓					
Botelho^24^	Assess adherence after checklist introduction	✓			✓	11	✓			✓	✓ATLS			
Botelho^25^	Evaluate of adherence	✓			✓	12	✓	✓		✓	✓ATLS		✓⁰	
Carter^26^	Identify factors related to delayed and omitted primary survey tasks		✓		✓	8	✓		✓		✓ATLS		✓inter-rater (r = 0.99, κ = 0.89)	
Civantos Fuentes^27^	Detect areas of improvement in simulation setting	✓		✓		11	✓	✓	✓			✓^30^		
Drost-de Klerck^29^	Evaluate performance regarding skills and competences during and after course	✓		✓		22	✓		✓				✓inter-rater T1, T2, T3 (ρ = 0.81, 0.61, 0.83)	
Falcone^30^	Evaluate effectiveness of multidisciplinary trauma team training		✓		✓	18	✓	✓	✓			✓^36^		✓
Gala^31^	Describe current performance of primary survey	✓			✓	11	✓		✓			✓^71^		✓
Gillman^32^	Course evaluation		✓	✓		14	✓	✓		✓	✓ATLS			
Glanville^33^	Evaluate the effectiveness of a learning program	✓			✓	19	✓	✓						
Gyedu^34^	Determine the achievement of key performance indicators during the initial assessment and management	✓			✓	18	✓	✓		✓	✓WHO			✓
Holcomb^36^	Validate advanced simulation as an evaluation tool for trauma team resuscitation skills		✓	✓		26	✓	✓	✓					✓
Hulfish^38^	Determine if cognitive aid checklist reduces omissions and speeds assessment time		✓	✓		14	✓	✓		✓		✓^69^		
Hultin^39^	Assess interrater reliability	✓		✓		10	✓	✓	✓			✓^36^	✓inter-rater (ICCs = 0.55 and 0.83)	
Hunt^65^	Characterize quality of resuscitation efforts and identify problem areas for educational interventions		✓	✓		16	✓	✓		✓	✓PALS, ATLS, TNCC		✓inter-rater (ICC=0.77, 95% CI^+^ 0.74–0.79)	✓
Innocenti^40^	Evaluate effectiveness of training program	✓		✓		23	✓	✓			✓Emergency Medicine Manual Oxford			
Jonsson^41^	Evaluate situational awareness training program on performance		✓	✓		10	✓		✓			✓^39^	✓^39^	
Kelleher^43^	Evaluate the effect of checklist on completion and timeliness of ATLS tasks		✓		✓	16	✓		✓			✓^69^		
Kelleher^44^	Evaluate effect checklist on deviations	✓			✓	6	✓		✓		✓ATLS			
Kelleher^45^	Analyse impact of team size on resuscitation task completion		✓		✓	24	✓		✓			✓^26^		
Kliem^47^	Investigate adherence	✓		✓		12	✓		✓				✓inter-rater (κ = 0.94)	
Koko^48^	Investigate adherence		✓	✓		17		✓		✓		✓^5^		
Linders^4^	Investigate adherence between video-based instruction versus conventional lecture	✓		✓		24	✓		✓		✓APLS		✓intra-rater (ICC=0.87, 95% CI 0.74–0.94)	
Lubbert^66^	Analyse team functioning and protocol deviations		✓		✓	26	✓	✓	✓		✓ATLS			
Maluso^51^	Determine the optimal number of team members in the initial evaluation		✓		✓	20	✓	✓	✓		✓ATLS			
Merriman^67^	Compare the use of teaching in clinical simulation or classroom	✓		✓		21	✓			✓	✓ALERT & ERC			
Nadel^52^	Evaluate effectiveness of an educational intervention on knowledge, technical skills and confidence	✓		✓		5	✓		✓		✓PALS			
O’Connell^53^	Evaluate effect of family presence on ATLS task performance		✓		✓	7	✓		✓			✓^26^	✓^26^	
Oakley^68^	Determine the ability of video review to identify management errors		✓		✓	12	✓		✓		✓ATLS		✓⁰	
Olgers^5^	Study completeness	✓			✓	26	✓							
Parsons^69^	Test checklist effectiveness		✓	✓		15	✓	✓	✓			✓^26^		✓
Peran^54^	Validate cognitive aid tool	✓		✓		36	✓	✓					✓⁰	✓
Pringle^55^	Assess effectiveness of a trauma course	✓		✓		11	✓		✓	✓				
Ritchie^57^	Assess utility of video review in assessing trauma team performance		✓		✓	20	✓	✓	✓			✓^72^		
Spanjers- Berg^59^	Analyse protocol compliance		✓		✓	29	✓	✓	✓					
Stayt^70^	Compare the use of teaching in clinical simulation or classroom	✓		✓		21	✓					✓^67^		
Tsang^61^	Assess protocol compliance		✓		✓	11	✓	✓			✓ATLS			
Wurster^62^	Evaluate competency of assessment physician		✓		✓	14	✓		✓				✓inter-rater (κ = 0.84, 95% CI 0.79–0.90)	✓
Yan^63^	Investigate the impact of rapid cycle deliberate practice on skill retention	✓			✓	8	✓					✓^31^		

*ICC: Intraclass Correlation Coefficient, κ: Kappa, r: Pearson’s correlation coefficient, ρ: Spearman’s Rho. ^+^95%-CI: 95% Confidence Interval was only provided if reported in the study.

⁰The study indicated that interrater was performed, but results were not reported.

Forty-three studies reported about adherence to the ABCDE approach, 22 in simulation setting, 21 in clinical practice (Table 1). The number of ABCDE assessments per study varied from 10 to 437.

#### Simulation setting

Overall adherence varied from 29% to 35% pre-intervention^29^, ^70^ and from 65% to 97% post-intervention (simulation training or course).^29^, ^55^ The frequency with which specific ABCDE items were assessed, varied from 80% for assessment of airway and breathing to 20% for stabilization of the cervical spine in trauma setting.^65^ Pulse oximetry and blood pressure measurement were performed in all simulations (100%), in contrast to pupillary examination (31%) and Glasgow Coma Scale (5%).^27^ Two studies investigated adherence for the separate domains (A, B, C, D, and E).^4^, ^47^ The highest and lowest adherence was measured in domain A (100%) and E (0%), respectively. Several studies reported influencing factors: the use of a cognitive aid tool^54^ or a handheld checklist resulted in higher adherence.^38^, ^69^ The type of healthcare professional might also impact adherence: physicians scored higher than nurses.^4^ Simulation training programs resulted in improved adherence both directly after the course and in the longer term (4 months to two years later) in some,^29^, ^40^ but not in all studies.^64^ According to three studies, teaching method influenced adherence.^4^, ^67^, ^70^ Virtual reality can be used as instruction method to teach students the ABCDE approach in the right order.^21^, ^22^

Time to completion of the ABCDE approach varied from within two minutes^38^, ^52^, ^55^ up to six minutes.^27^, ^36^, ^54^ The use of a cognitive aid tool or checklist did not influence assessment time.^38^, ^54^

#### Clinical practice

In clinical studies, overall adherence towards the ABCDE approach ranged from 18% to 84%.^5^, ^25^ One study showed that the ABCDE approach was used in the minority (33%) of unstable patients and in only 3% of presumably stable patients in the emergency department.^5^ The frequency with which specific ABCDE items were assessed by participants varied widely, for example: airway patency from 76% to 100%, respiratory rate from 7% to 100%, and measurement of temperature from 0% to 100%.^5^, ^17^, ^26^, ^33^, ^43^, ^59^, ^61^, ^62^ One study showed that incomplete adherence involved basic ABCDE assessment principles such as omission of chest auscultation (44%) and central capillary refill time assessment (66%).^68^ Higher adherence was observed in the presence of an identified team leader^35^, ^51^, ^61^; lower adherence with increased injury severity.^25^ The number of team members influenced adherence, with an optimum of seven team members.^45^, ^51^ The use of a checklist,^24^ training in situational awareness,^41^ nor family presence^53^ influenced adherence, neither did speciality.^25^ Reported reasons for not applying the ABCDE approach were that clinical impression, vital signs or reason for visiting the emergency department did not indicate instability of the patient.^5^

Time to start the ABCDE approach after patient arrival to the emergency department varied from two minutes to 57 min and was significantly decreased by increasing triage code.^5^, ^24^ Time to complete the ABCDE approach varied from within 2 min to more than 30 min.^5^, ^17^, ^26^, ^31^, ^35^, ^53^, ^57^, ^59^, ^60^, ^63^, ^66^ Time needed to assess all domains of the ABCDE approach depended on the condition of the patient (e.g. injury severity)^51^ and the number of therapeutic interventions performed.^31^ The use of a handheld checklist increased the speed of the assessment of vital signs, resulting in a faster completion of the ABCDE approach.^43^

3) **Other outcomes**
a. ***Professional outcomes***

In 18 studies professional outcomes, such as knowledge and confidence were reported (simulation setting (n = 15), clinical practice (n = 3) (Table 1)).

#### Simulation setting

Attending a simulation course increased participants’ confidence in adequately assessing critically ill patients.^18^, ^19^, ^28^, ^36^, ^37^, ^52^ There was no correlation between self-reported confidence or self-efficacy and ABCDE performance.^67^, ^70^ The use of a displayed primary survey checklist resulted in a slight (not significant) increase in mental demand and effort in team leaders compared to no checklist.^38^ An interdisciplinary paediatric trauma simulation program resulted in improved overall assessment scores including teamwork.^64^

#### Clinical practice

Scores on a theoretical knowledge test of the ABCDE approach varied among healthcare professionals (mean test scores: 80.1%, SD 12.2 and 52.9%, SD 12.2).^16^, ^58^ Type of department, profession category and age significantly influenced test scores. Nurses reported they felt most confident in the assessment and management of the circulation (88.2%) compared to airway (58.8%) and breathing (40.5%).^56^ The application of the ABCDE approach facilitated determination of a diagnosis and enhancing the decision to administer oxygen or intravenous fluids.^19^

b. ***Team outcomes***

Reported team outcomes concerned communication, leadership and teamwork.^17^, ^23^, ^35^, ^42^, ^51^, ^57^, ^61^, ^64^, ^66^ While applying the ABCDE approach, understandable communication (i.e. clear questions or instructions) between team members varied from 6% to 70%.^23^ Errors in team organization (unclear or inefficient team leader, unorganized resuscitation, not working according to protocol, and discontinuous supervision of the patient) led to significantly more deviations from the primary survey than when team organization was clearly defined.^66^ A paediatric trauma simulation program resulted in improved scores for teamwork.^64^

c. ***Patient outcomes***

Only two studies reported patient outcomes. One study showed that patients with a higher, although not significant, mean adherence to the ABCDE had shorter length of stay at the emergency department.^62^ The other presented that healthcare professionals scoring higher in ABCDE adherence ordered fewer CT scans, with no difference in patient mortality.^25^

## Discussion

This review identified 57 studies that reported assessment, adherence, and other outcomes related to application of the ABCDE approach in clinical practice and simulation setting. To our knowledge, this is the first literature review focusing on the ABCDE approach. Reviews about Advanced Life Support (ALS) guidelines and Advanced Trauma Life Support (ATLS) courses exist, but did not describe outcomes related to the ABCDE approach nor the primary survey specifically.^73^, ^74^, ^75^

1) **ABCDE assessment tools**

A reason for the large variation in content of ABCDE assessment tools might be that the assessment tools were developed with various goals. Some tools focused on details on the content of and adherence to the ABCDE approach. Other tools were developed to study the ABCDE approach in a broader perspective, for example, to answer research questions about team outcomes and education. Therefore, studies might have chosen a limited number of items in their assessment tool for practical reasons (e.g. to ease scoring). Another potential explanation might be that interprofessional disagreement exists about the most important ABCDE items. Moreover, several tools included the action items following the ABCDE assessment in their assessment tools (Table 2). These action items might have different functions, for example, ‘consider ordering blood’ was designed to test higher level decision making.^69^ While the ABCDE approach itself is strictly a structured method to assess a patient, it should be linked to clinical decision-making including appropriate management, i.e. actions, in order to improve a patient’s condition.

2) **Adherence to the ABCDE approach**

A wide variation in adherence is evident. Considering the variation in content of assessment tools, but also in context, setting and participants, fair comparison is not possible and the actual performance of professionals with regards to the ABCDE remains largely unknown. However, most studies showed suboptimal adherence. In this review, we identified factors influencing adherence which might reveal opportunities for improvement in every department treating (potentially) critically ill patients. For example, to assign a team leader in your daily team and to regularly attend simulation training can already lead to increased adherence.^35^, ^51^, ^61^ Nurses scored low on adherence and knowledge, suggesting a tailored approach to benefit these healthcare professionals.^4^, ^16^, ^58^ Thereby, as with all skills, practice is the key for retention of skills. For the ABCDE approach itself this has not been investigated yet, but it is known that ALS knowledge and skills decay by six months to one year after training and that skills decay faster than knowledge.^73^ The ABCDE approach is a quick and simple, however valuable tool as one assessment enables healthcare professionals to collect relevant information about the current condition of the patient, and repeated assessments provide trend monitoring to prioritize the associated treatment. Performing only a first clinical impression or collecting some vital signs without a structure, might risk missing a critically ill patient or a significant deterioration. Although adherence indicates how precise the ABCDE algorithm is performed, in a real patient the actions following the assessment are as important. A patient will not improve as a result of perfect adherence per se, but hopefully as a result of the actions based on the assessment. Even though some studies included actions in their assessment tools, no study investigated the associated actions separately.

3) **Outcomes**

It is remarkable that only two studies in this review reported the ABCDE approach in association with patient outcomes.^25^, ^62^ Two other studies were interested in patient outcomes, but could not report results as result of underpowering and lack of relevant outcomes in the studies.^24^, ^59^ For the ATLS, of which the primary survey, and thus the ABCDE approach is an important element, it is also known that evidence confirming reduction of morbidity or mortality is still lacking.^74^, ^75^ If the goal of the ABCDE approach is to identify potentially life-threatening conditions followed by lifesaving actions, failure to complete the ABCDE approach in a complete and efficient manner might influence patient outcomes. Setting up a randomized controlled trial comparing the ABCDE approach versus no structured approach in the assessment of a real patient is not possible given the wide adoption of the ABCDE approach. Therefore, detailed study of adherence, the factors influencing this adherence and the association of this adherence to clinical outcomes might shed light on this hitherto largely unexplored area.

### Strengths and limitations

This scoping review, performed in concordance with the JBI guidelines, is an important and new contribution to the existing knowledge about the ABCDE approach as it identified research regarding the approach and its outcomes. Strengths are the use of a broad search strategy in multiple databases and the structured scoping approach including two researchers independently performing study selection and data extraction. Foremost, the heterogeneity of the included studies stands out as it limits comparison of the results.

### Knowledge gaps and implications

Based on this scoping review, the following knowledge gaps were identified:

• **No uniformity in reported assessment tools.** Standardization of the assessment tools, along with appropriate validation evidence, is needed. A universal ABCDE approach and subsequently a universal assessment tool could be part of that process.
• **Effectiveness of application remains unknown**. Randomized controlled trials comparing the ABCDE approach to no structure do not exist and data on patient outcomes were scarce. Reported adherence varied widely and was measured inconsistently. Although perfect adherence does not necessarily guarantee improvement of patient outcomes, decision making and calling for appropriate actions following the ABCDE assessment might be related to this adherence. As such, uniformity and clarity in reporting on the ABCDE approach and its assessment is needed as a first step towards potential improvement of patient outcomes. A consensus-based core outcome set (COS) should be developed, consisting of outcome measures which are easy to use, demonstrate suitable measurement properties to evaluate application of the ABCDE approach, facilitating comparison.^76^, ^77^ For future researchers publishing about the ABCDE approach, we recommend providing detailed information about the assessment tools and outcome measurements used.
• **Teaching and implementation underexplored.** There is a lack of understanding regarding teaching methods to improve application and, in particular, adherence in clinical care. However, a reliable and valid assessment tool is needed to further investigate this. Teaching is mainly focused on simulation training, but learning should be continued afterwards in daily clinical care. Therefore, workplace learning is essential to continue the learning process and thereby improving implementation and adherence.

## Conclusion

This scoping review showed that a large variety of ABCDE assessment tools exists. Adherence varied widely among included studies. The effects of the ABCDE approach on patient outcomes are yet to be established.

## CRediT authorship contribution statement

**Laura J. Bruinink:** Writing – original draft, Methodology, Investigation, Formal analysis. **Marjolein Linders:** Writing – original draft, Methodology, Investigation, Formal analysis, Conceptualization. **Willem P. de Boode:** Writing – review & editing, Conceptualization. **Cornelia R.M.G. Fluit:** Writing – review & editing. **Marije Hogeveen:** Writing – review & editing, Supervision, Methodology, Conceptualization.

## Declaration of competing interest

The authors declare that they have no known competing financial interests or personal relationships that could have appeared to influence the work reported in this paper.
