# Supplementary material for: The ABCDE approach in critically ill patients: A scoping review of assessment tools, adherence and reported outcomes
Source: Resusc Plus. 2024 Sep 19;20:100763. doi: 10.1016/j.resplu.2024.100763 (PMC11437753; doi:10.1016/j.resplu.2024.100763)
Supplement: Supplementary Data 1 [file mmc1.pdf]

# Supplementary Materials

## Appendix A: PRISMA Scr-Checklist

| SECTION | ITEM | PRISMA-ScR CHECKLIST ITEM | REPORTED ON PAGE # |
|---|---|---|---|
| **TITLE** | | | |
| Title | 1 | Identify the report as a scoping review. | 1 |
| **ABSTRACT** | | | |
| Structured summary | 2 | Provide a structured summary that includes (as applicable): background, objectives, eligibility criteria, sources of evidence, charting methods, results, and conclusions that relate to the review questions and objectives. | 1 |
| **INTRODUCTION** | | | |
| Rationale | 3 | Describe the rationale for the review in the context of what is already known. Explain why the review questions/objectives lend themselves to a scoping review approach. | 2 |
| Objectives | 4 | Provide an explicit statement of the questions and objectives being addressed with reference to their key elements (e.g., population or participants, concepts, and context) or other relevant key elements used to conceptualize the review questions and/or objectives. | 2 |
| **METHODS** | | | |
| Protocol and registration | 5 | Indicate whether a review protocol exists; state if and where it can be accessed (e.g., a Web address); and if available, provide registration information, including the registration number. | 2 |
| Eligibility criteria | 6 | Specify characteristics of the sources of evidence used as eligibility criteria (e.g., years considered, language, and publication status), and provide a rationale. | 3 |
| Information sources* | 7 | Describe all information sources in the search (e.g., databases with dates of coverage and contact with authors to identify additional sources), as well as the date the most recent search was executed. | 4 |
| Search | 8 | Present the full electronic search strategy for at least 1 database, including any limits used, such that it could be repeated. | Appendix B |
| Selection of sources of evidence† | 9 | State the process for selecting sources of evidence (i.e., screening and eligibility) included in the scoping review. | 4 |
| Data charting process‡ | 10 | Describe the methods of charting data from the included sources of evidence (e.g., calibrated forms or forms that have been tested by the team before their use, and whether data charting was done independently or in duplicate) and any processes for obtaining and confirming data from investigators. | 4 |
| Data items | 11 | List and define all variables for which data were sought and any assumptions and simplifications made. | 4<br>Appendix D |
| Critical appraisal of individual sources of evidence§ | 12 | If done, provide a rationale for conducting a critical appraisal of included sources of evidence; describe the methods used and how this information was used in any data synthesis (if appropriate). | 4 |

| SECTION | ITEM | PRISMA-ScR CHECKLIST ITEM | REPORTED ON PAGE # |
|---|---|---|---|
| Synthesis of results | 13 | Describe the methods of handling and summarizing the data that were charted. | 5-6 |
| **RESULTS** | | | |
| Selection of sources of evidence | 14 | Give numbers of sources of evidence screened, assessed for eligibility, and included in the review, with reasons for exclusions at each stage, ideally using a flow diagram. | 5 |
| Characteristics of sources of evidence | 15 | For each source of evidence, present characteristics for which data were charted and provide the citations. | 5 |
| Critical appraisal within sources of evidence | 16 | If done, present data on critical appraisal of included sources of evidence (see item 12). | Not applicable |
| Results of individual sources of evidence | 17 | For each included source of evidence, present the relevant data that were charted that relate to the review questions and objectives. | 5-9 |
| Synthesis of results | 18 | Summarize and/or present the charting results as they relate to the review questions and objectives. | 5-9 |
| **DISCUSSION** | | | |
| Summary of evidence | 19 | Summarize the main results (including an overview of concepts, themes, and types of evidence available), link to the review questions and objectives, and consider the relevance to key groups. | 9-12 |
| Limitations | 20 | Discuss the limitations of the scoping review process. | 11 |
| Conclusions | 21 | Provide a general interpretation of the results with respect to the review questions and objectives, as well as potential implications and/or next steps. | 12 |
| **FUNDING** | | | |
| Funding | 22 | Describe sources of funding for the included sources of evidence, as well as sources of funding for the scoping review. Describe the role of the funders of the scoping review. | 13 |

## Appendix B: Search strategy (MEDLINE)

ABCDE[tiab] OR airway breathing circulation disability exposure OR airway breathing circulation disability environment OR ((Airway[tiab] OR airway management[mesh] OR airway obstruction[mesh] OR Breathing[tiab] OR dyspnea[mesh] OR dyspnea[tiab] OR dyspnoea[tiab] OR respiratory insufficiency[mesh] OR respiratory insufficien*[tiab] OR respiratory rate[mesh] OR respiratory rate[tiab] OR oxygen saturation[tiab]) AND (Circulation[Text Word] OR flow, regional blood[mesh] OR blood pressure[mesh] OR blood pressure[tiab] OR heart rate[mesh] OR heart rate[tiab] OR capillary refill[tiab]) AND (Disability[tiab] OR neurological examination[mesh] OR neurological examination[tiab] OR neurologic examination[tiab] OR glasgow coma scale[tiab] OR GCS[tiab] OR AVPU[tiab] OR alert verbal pain unresponsive OR blood glucose[mesh] OR glucose[tiab]) AND (Exposure[tiab] OR environment[tiab] OR body temperature[mesh] or temperature[tiab]))

OR

(Primary survey[tiab] OR primary assessment[tiab] OR initial assessment[tiab]) AND (Care, Life support[mesh] OR life support[tiab] OR Resuscitation[mesh] OR resuscitation[tiab] OR care, critical[mesh] OR critical care[tiab] OR critical illness[mesh] OR critical illness OR emergency treatment[mesh] OR emergency medicine[mesh] OR emergency hospital[mesh] OR emergency medical service[mesh] OR emergency[tiab] OR emergencies[tiab])

## Appendix C: Studies excluded following full-text review

| | First author (year) | Title | Reason for exclusion |
|---|---|---|---|
| 1 | Abu-Zidan (2014) | Factors Affecting Success Rate of Advanced Trauma Life Support (ATLS) Courses | Wrong concept |
| 2 | Ahmad (2022) | The effect of implementing adult trauma clinical practice guidelines on outcomes of trauma patients and health care providers | Wrong concept |
| 3 | Allana (2020) | Delivery of a primary trauma care course in a developing country | Full text not available (conference abstract) |
| 4 | Ali (1999) | Comparison of Performance of Interns Completing the Old (1993) and New Interactive (1997) Advanced Trauma Life Support Courses | Wrong concept |
| 5 | Allorto (2012) | An audit of the quality of initial trauma resuscitation in a regional hospital | Full text not available (conference abstract) |
| 6 | Amiri (2013) | Two-day primary trauma care workshop: early and late evaluation of knowledge and practice | Wrong concept |
| 7 | Anderson (2018) | Development of a Comprehensive Trauma Training Curriculum for the Resource-Limited Environment | Wrong concept |
| 8 | Angotti (2020) | Electronic trauma resuscitation documentation and decision support using T6 Health Systems Mobile Application: A combat trauma center pilot program | Wrong concept |
| 9 | Babu (2020) | Participants' perception of the AIIMS Trauma Assessment and Management (ATAM) course for management of polytrauma: A multi-institutional experience from India | Wrong concept |
| 10 | Baer (2017) | Improving interdepartmental trauma evaluation and resuscitation through mock in situ trauma review and debriefing | Full text not available (conference abstract) |
| 11 | Becher (1983) | The initial assessment and care of the patient with multiple trauma | Full text not available |
| 12 | Betlehem (2009) | The characteristics of ambulance officer students' performance in ALS examination | Full text in Hungary |
| 13 | Berg (2023) | Developing a virtual reality (VR) application for practicing the ABCDE approach for systematic clinical observation | Wrong concept |
| 14 | Biban (2014 | Management of red codes in the paediatric emergency room | Full text not available (conference abstract) |
| 15 | Bidhendi (2021) | Evaluating implementation of WHO Trauma Care Checklist vs. modified WHO checklist in improving trauma patient clinical outcomes and satisfaction | Wrong concept |
| 16 | Birkebaek (2021) | Implementation of ABC-team at regional hospital West Jutland | Full text not available (conference abstract) |
| 17 | Boeck (2018) | The development and implementation of a layperson trauma first responder course in La Paz, Bolivia: A pilot study | Wrong population |
| 18 | Bradley (2017) | Quality of communication during transfer to definitive care in a provincial trauma system: A review of 100 transfers | Wrong concept |
| 19 | Briggs (2015) | The Role of Nontechnical Skills in Simulated Trauma Resuscitation | Wrong concept |
| 20 | Brigham (2022) | Pediatric trauma care: Strengths and weaknesses at a level one pediatric trauma center | Full text not available (conference abstract) |
| 21 | Brown (2023) | Maximizing the golden hour in critically ill patients: assessment gaps at the time of admission | Full text not available |
| 22 | Bruno (2015) | Simulation as a tool for improving acquisition of neonatal resuscitation skills for obstetric residents | Wrong concept |
| 23 | Campbell (2021) | Use of simulation to develop leadership | Full text not available (conference abstract) |
| 24 | Carbery (2016) | 'Predict, assess, treat': Developing clinical skills in recognizing and responding to clinical deterioration: Outcomes of a scenario based clinical skills program for clinical staff in a tertiary referral acute hospital setting | Full text not available (conference abstract) |

| | | | |
|---|---|---|---|
| **25** | Carling (2010) | Are graduate doctors adequately prepared to manage acutely unwell patients? | Wrong concept |
| **26** | Carrington (2009) | Recognition and assessment of critical illness | Background article |
| **27** | Celso (2006) | A Systematic Review and Meta-Analysis Comparing Outcome of Severely Injured Patients Treated in Trauma Centers Following the Establishment of Trauma Systems | Wrong concept |
| **28** | Chamberlain (2022) | Pediatric code blue event analysis: performance of non-acute health-care providers | Wrong concept |
| **29** | Chowdhury (2022) | Trauma resuscitation training: An evaluation of nurses' knowledge | Wrong concept |
| **30** | Chung (2018) | The educational impact of web-based and face-to-face patient deterioration simulation programs: An interventional trial | Wrong concept |
| **31** | Cleaver (2021) | Evaluation of a new rapid assessment and treatment (RAT) tablet app for Emergency Department (ED) nurses: Is earlier identification of investigations and treatments feasible? | Wrong concept |
| **32** | Cleugh (2017) | Management of the multiply injured child | Background article |
| **33** | Connell (2016) | The effectiveness of education in the recognition and management of deteriorating patients: A systematic review | Wrong concept |
| **34** | Cooper (2010) | Managing the deteriorating patient in a simulated environment: nursing students' knowledge, skill and situation awareness | Wrong concept |
| **35** | Danioth (2023) | Resuscitation room interventions in non-traumatological patients and their evidence | Wrong concept |
| **36** | Djärv (2023) | Reversible causes: After three decades with 4H4Ts, might we be ready for the generic ABCDE approach? | Letter to the editor |
| **37** | Douglas (2010) | Improving trauma care in India: a recommendation for the implementation of ATLS training for emergency department medical officers | Wrong concept |
| **38** | Driscoll (1992) | Variation in trauma resuscitation and its effect on patient outcome | Wrong concept |
| **39** | Fernández (2017) | The Validity of the Pediatric Assessment Triangle as the First Step in the Triage Process in a Pediatric Emergency Department | Wrong concept |
| **40** | Farahmand (2016) | Distance Learning Can Be as Effective as Traditional Learning for Medical Students in the Initial Assessment of Trauma Patients | Wrong concept |
| **41** | Fern (2019) | ABCDE approach to victims by lifeguards: How do they manage a critical patient? A cross sectional simulation study | Wrong population |
| **42** | Gascon (2022) | Mixed methods approach to understanding the influence of changes in successive versions of the advanced trauma life support training program on student performance | Wrong concept |
| **43** | Granström (2019) | Patient experiences of initial trauma care | Background article |
| **44** | Gyedu (2023) | Care of Injured Children Compared to Adults at District and Regional Hospitals in Ghana and the Impact of a Trauma Intake Form: A Stepped-Wedge Cluster Randomized Trial | Wrong concept |
| **45** | Gyedu (2023) | Standardized trauma intake form with clinical decision support prompts improves care and reduces mortality for seriously injured patients in non-tertiary hospitals in Ghana: stepped-wedge cluster randomized trial | Wrong concept |
| **46** | Halbert (2015) | Improving patient care through paediatric simulation and multi-disciplinary resuscitation training based upon previous serious incidents at a district general hospital | Full text not available (conference abstract) |
| **47** | Hart (2018) | Simulation for Assessment of Milestones in Emergency Medicine Residents | Wrong concept |
| **48** | Haske (2017) | Subjective safety and self-confidence in prehospital trauma care and learning progress after trauma-courses: part of the prospective longitudinal mixed-methods EPPTC trials | Wrong context |
| **49** | Hardenberg (2020) | Evaluating Impact of Repeated Exposure to High Fidelity Simulation: Skills Acquisition and Stress Levels in Postgraduate Critical Care Nursing Students | Wrong concept |

| | | | |
|---|---|---|---|
| **50** | Herron (2019) | Effect of case study versus video simulation on nursing students' satisfaction, self-confidence, and knowledge: a quasi-experimental study | Wrong concept |
| **51** | Hogg (2016) | The effects of an enhanced simulation programme on medical students' confidence<br>responding to clinical deterioration | Wrong concept |
| **52** | Hovenkamp (2018) | The satisfaction regarding handovers between ambulance and emergency department nurses: an observational study | Wrong concept |
| **53** | Jafri (2021) | A Microdebriefing Crisis Resource Management Program for Simulated Pediatric Resuscitation in a Community Hospital | Wrong concept |
| **54** | Jansen (2023) | Indications and measures of medical emergency teams: a retrospective evaluation of in-hospital emergency operations of the German Resuscitation Register | Full text not available |
| **55** | Jensen (2015) | Emergency team calls for critically ill non-trauma patients in the emergency department: an observational study | Wrong concept |
| **56** | Jessel (2022) | Major Obstetric Trauma Simulation -Learning from Experience | Full text not available (conference abstract) |
| **57** | Jevon (2010) | ABCDE: The assessment of the critically ill patient | Background article |
| **58** | Kantonen (2015) | Impact of an ABCDE team triage process combined with public guidance on the division of work in an emergency department | Wrong concept |
| **59** | Katzenschlager (2022) | Implementation of hyperspectral imaging in a trauma resuscitation room: a randomized controlled trial | Wrong concept |
| **60** | Keilman (2021) | Enhancing paediatric resuscitation team performance: targeted simulation-based team leader training | Wrong concept |
| **61** | Kelleher (2013) | Factors associated with patient exposure and environmental control during pediatric trauma resuscitation | Wrong concept |
| **62** | Kelley (2021) | Excellence in Communication and Emergency Leadership (ExCEL): Pediatric Primary and Secondary Survey in Trauma Workshop for Residents | Wrong concept |
| **63** | Kennedy (2001) | The ATLS course, a survey of 228 ATLS providers | Wrong concept |
| **64** | Lampi (2013) | Triage performance of Swedish physicians usingthe ATLS algorithm in a simulated mass casualtyincident: a prospective cross-sectional survey | Wrong concept |
| **65** | Lansink (2007) | Do designated trauma systems improve outcome | Wrong concept |
| **66** | Large (2021) | National evaluation of trauma teaching for students (NETTS) | Wrong concept |
| **67** | Lashoher (2017) | Implementation of the World Health Organization Trauma Care Checklist Program in 11 Centers Across Multiple Economic Strata: Effect on Care Process Measures | Wrong concept |
| **68** | Lekoudis (2015) | A multidisciplinary in situ simulation program in obstetric emergencies: 'Working and learning together' | Full text not available (conference abstract) |
| **69** | Levison (1982) | Initial assessment and resuscitation | Full text not available |
| **70** | Lewis (1984) | Initial assessment and resuscitation | Full text not available |
| **71** | Liaw (2015) | Designing and Evaluating an Interactive Multimedia Web-Based Simulation for Developing Nurses' Competencies in Acute Nursing Care: Randomized Controlled Trial | Wrong concept |
| **72** | Lima (2019) | Multiple victims incident simulation: training professionals and university teaching | Wrong context |
| **73** | Linders (2020) | Factors influencing healthcare professionals' adherence to the systematic ABCDE approach: A qualitative mixed-method study | Full text not available (conference abstract) |
| **74** | Lo (2014) | Evaluation of an advanced Trauma life support course in Taiwan | Wrong concept |
| **75** | Lodemann (2022) | Process Modelling of ABCDE Primary Survey in Trauma Resuscitations: A Crucial First Step for Agent-Based Simulation Modelling of Complex Team-Based Clinical Processes | Wrong concept |
| **76** | Logarajah (2014) | An integrated ABCDE approach to managing medical emergencies using CRM principles | Wrong concept |
| **77** | Marikar (2017) | Graphics in sim training (GIST): A technology aid for performance assessment in paediatric emergency department in-situ simulation | Full text not available (conference abstract) |

| | | | |
|---|---|---|---|
| **78** | Martinho (2024) | Observational Study About the Impact of Simulation Training of Non-Technical Skills on Teamwork: Towards a Paradigm Shift in Undergraduate Medical Training | Wrong concept |
| **79** | Mayo (2017) | Undertaking an accurate and comprehensive assessment of the acutely ill adult | Full text not available |
| **80** | McCarthy (2015) | A feasible, acceptable and effective way to teach health care workers in low- and middle-income countries a method to manage acutely ill obstetric women | Wrong concept |
| **81** | Mehra (2018) | Common Pediatric Medical Emergencies in Office Practice | Wrong context |
| **82** | Mohammad (2013) | Educational and Clinical Impact of Advanced Trauma Life Support (ATLS) Courses: A Systematic Review | Wrong concept |
| **83** | Mould-Millman (2015) | Assessing trauma care knowledge, attitudes, and skills in African prehospital providers | Wrong concept, wrong context |
| **84** | Munroe (2016) | A structured framework improves clinical patient assessment and nontechnical skills of early career emergency nurses: a pre–post study using full immersion simulation | Wrong concept |
| **85** | Nguyen (2021) | Assessment of Emergency Medicine Resident Performance in a Pediatric In Situ Simulation Using Multi-Source Feedback | Wrong concept |
| **86** | Nussberger (2022) | Pediatric emergency room training: A novel, low-threshold approach | Full text not available (conference abstract) |
| **87** | Ologunde (2017) | Do trauma courses change practice? A qualitative review of 20 courses in East, Central and Southern Africa | Background article |
| **88** | Parker (2017) | The Upstart ABC project-low fidelity simulation training in Cambodia | Full text not available (conference abstract) |
| **89** | Piquette (2021) | Compétences des résidents avant et après de courts stages à l'unité de soins intensifs : une étude pilote observationnelle multicentriqu | Wrong concept |
| **90** | Preston (2009) | Assessing advanced life support (ALS) competence: Victorian practices | Wrong concept |
| **91** | Ruesseler (2010) | Increased authenticity in practical assessment using emergency case OSCE stations | Wrong concept |
| **92** | Rybinski (2019) | The impact of "pause and debrief" simulation training on acquisition of knowledge in pre-clinical medical education | Full text not available (conference abstract) |
| **93** | Rybinski (2019) | Understanding how a new debriefing model enhances learning in novice learners | Full text not available (conference abstract) |
| **94** | See (2014) | Effectiveness of a Patient Education Intervention in Enhancing Patients' Self-efficacy to Recognise and Report Acute Deterioration: a Pilot Randomised Controlled Trial | Wrong concept |
| **95** | Shrestha (2018) | Improving knowledge, skill and confidence of novice medical doctors in trauma management with principles of ABCDE | Wrong concept |
| **96** | Siebers (2009) | Emergency room management of severely injured patients | Wrong concept |
| **97** | Simoes (2014) | Trauma leagues: an alternative way to teach trauma surgery to medical students | Wrong concept |
| **98** | Simpson (2012) | Quality care in pediatric trauma | Wrong concept |
| **99** | Sluiter (2018) | ABCDE, what can you do with it? | Background article |
| **100** | Sokol (2015) | Prehospital interventions in severely injured pediatric patients: Rethinking the ABCs | Wrong context |
| **101** | Solevag (2012) | The effect of quality improvement initiatives as measured by nurses' perceptions in a department of children and adolescents | Full text not available (conference abstract) |
| **102** | Steadman (2006) | Simulation-based training is superior to problem-based learning for the acquisition of critical assessment and management skills | Wrong concept |
| **103** | Stuart (2015) | Can simulation based introduction for new employees improve patient safety? | Full text not available (conference abstract) |
| **104** | Suda (2022) | Delayed diagnosed trauma in severely injured patients despite guidelines-oriented emergency room treatment: there is still a risk | Wrong concept |
| **105** | Sumritrin (2021) | Implementation of clinical nursing practice guideline for treatment of multiple injury patients admitted to Emergency Department | Wrong concept |

| | | | |
|---|---|---|---|
| **106** | Tarradas (2017) | Analysis of the implantation of an immediate life support program | Full text not available (conference abstract) |
| **107** | Thilakasiri (2019) | Implementing near-peer learning: a resuscitation skills development module for medical students in middle income countries | Full text not available (conference abstract) |
| **108** | Thim (2012) | Initial assessment and treatment with the Airway, Breathing, Circulation, Disability, Exposure (ABCDE) approach | Background article |
| **109** | Turner (2006) | The effect of the Advanced Paediatric Life Support course on perceived self-efficacy and use of resuscitation skills | Wrong concept |
| **110** | Turner (2009) | The effect of the APLS-course on self-efficacy and its relationship to behavioural decisions in paediatric resuscitation | Wrong concept |
| **111** | Van Maarseveen (2020) | Effects of the application of a checklist during trauma resuscitations on ATLS adherence, team performance, and patient-related outcomes: a systematic review | Individual studies were assessed and one study (Kelleher 2013) was included |
| **112** | Van Olden (2003) | Trauma resuscitation time | Wrong concept |
| **113** | Viggers (2013) | Improving self-perceived competence in medical students using simulation | Full text not available (conference abstract) |
| **114** | Von Atzingen (2008) | Elaboration and application of an evaluation instrument in the immediate postoperative period, based on the Advanced Trauma Life Support protocol | Wrong concept |
| **115** | Walsh (1989) | The effectiveness of the Advanced Trauma Life Support system in a mass casualty situation by non-trauma-experienced physicians: Grenada 1983 | Background article |
| **116** | Wiggins (2020) | Using Simulation to Train Medical Units for Deployment | Wrong concept |
| **117** | Wilson (2011) | Trauma and burns in children | Background article |
| **118** | Wing (2020) | Excellence in Communication and Emergency Leadership (ExCEL): Pediatric First 5 Minutes Workshop for Residents | Wrong concept |
| **119** | Wood (1995) | Use of video recorders in auditing initial assessment times | Wrong concept |
| **120** | Woollard (2004) | The ABC of community emergency care: 5 assessment and identification of paediatric primary survey positive patients | Background article |
| **121** | Wu (2018) | Development of an ABLS (advanced burn life support) course-based training program for nurses in China | Full text not available (conference abstract) |
| **122** | Yurt (1992) | Triage, initial assessment, and early treatment of the pediatric trauma patient | Background article |

## Appendix D: Data extraction instrument

| | |
|---|---|
| **Evidence source details and characteristics** | |
| Author | |
| Title | |
| Year of Publication | |
| Year of Data | |
| Journal | |
| Country | |
| Aim of study | |
| Outcomes (primary and secondary) | |
| Study design | |
| **Inclusion/Exclusion criteria** | |
| Population/Participants | |
| Concept | |
| Context<br>- Discipline | |
| **Results extracted from source of evidence** | |
| Key findings | |
| Adherence | |
| Professional outcomes | |
| Team outcomes | |
| Patient outcomes | |
| Other outcomes | |
| ABCDE algorithm / assessment tool:<br>- Number of items<br>- Video and/or live observation<br>- Assessment or action items<br>- Validation evidence | |
| References | |
